# Expanding the genetic toolbox of the obligate predatory bacterium *Bdellovibrio bacteriovorus* with inducible gene expression and CRISPR interference

**DOI:** 10.1093/femsml/uqaf021

**Published:** 2025-09-01

**Authors:** Charles de Pierpont, Benoît Derneden, Ophélie Remy, Géraldine Laloux

**Affiliations:** de Duve Institute, UCLouvain, Brussels, 1200, Belgium; de Duve Institute, UCLouvain, Brussels, 1200, Belgium; de Duve Institute, UCLouvain, Brussels, 1200, Belgium; de Duve Institute, UCLouvain, Brussels, 1200, Belgium; WEL Research Institute, Wavre, 1300, Belgium

**Keywords:** *Bdellovibrio bacteriovorus*, genetic tools, CRISPRi, IPTG-inducible gene expression, *ftsZ*, predatory bacteria

## Abstract

*Bdellovibrio bacteriovorus* is an obligate predatory bacterium that invades the periplasm of diderm prey bacteria, where it elongates and produces multiple daughter cells through nonbinary division. Investigating the molecular determinants of this lifecycle is challenging because deleting genes required for predation also impairs survival. Furthermore, the scarcity of robust conditional gene expression systems has restricted functional studies in this bacterium. Here, we address these limitations by expanding the genetic toolbox for *B. bacteriovorus*. First, we analysed the relative strength of a series of promoters, providing new resources to fine-tune gene expression. We then established an isopropyl β-D-1-thiogalactopyranoside (IPTG)-inducible expression system that can be activated during both the attack and growth phases of the predator. Finally, we designed a CRISPR interference (CRISPRi) module for IPTG-inducible gene knockdown, enabling rapid and targeted depletion. As a proof of principle, CRISPRi-mediated silencing of the cell curvature gene *bd1075* reproduced the straight phenotype of the deletion mutant. Likewise, depletion of the tubulin homologue FtsZ—which we showed is essential for *B. bacteriovorus* survival—blocked cell division within the first replicative cycle, yielding filamentous progeny still able of exiting the prey cell. This highlights the intriguing potential of uncoupling key cell cycle and predatory processes. Overall, these tools significantly broaden the scope of genetic manipulation in *B. bacteriovorus* and open new avenues for in-depth investigation of its noncanonical biology.

## Teaser

Inducible gene expression and CRISPR interference-mediated depletion in the predatory bacterium *Bdellovibrio bacteriovorus*.

## Introduction

**Figure 1.**
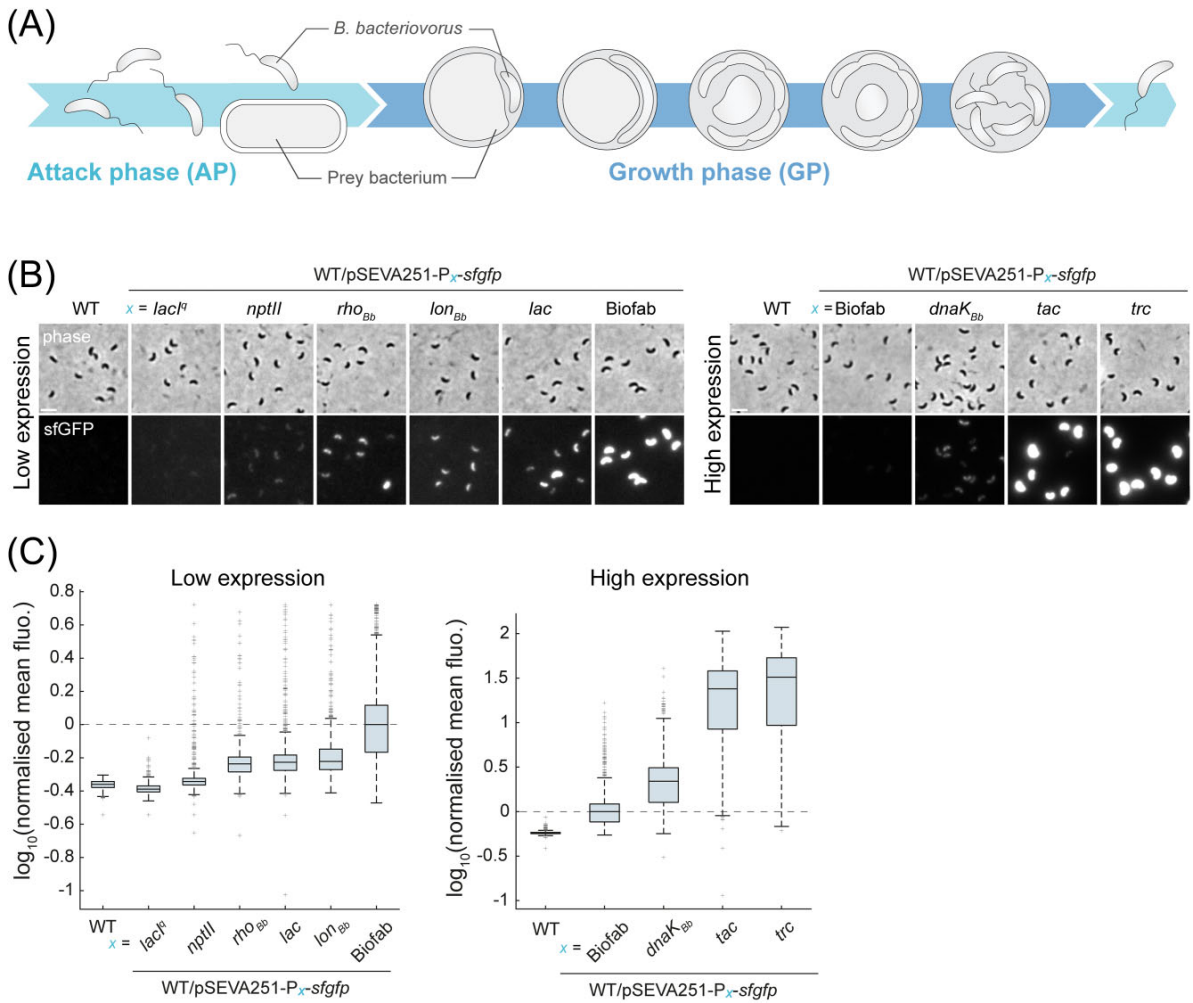
Relative strengths of a set of constitutive promoters in *B. bacteriovorus*. (A) Schematics of the *B. bacteriovorus* predatory lifecycle, including the nonreplicative AP and the replicative GP inside a diderm prey bacterium. In this study, *Escherichia coli* is used as prey. (B) Representative phase contrast and sfGFP fluorescence images for wild-type *B. bacteriovorus* HD100 (WT) and *B. bacteriovorus* strains carrying the pSEVA251 plasmid with the *sfgfp* reporter under control of the indicated promoters. Note that P*_lac_*, P*_tac_*, and P*_trc_* promoters in these reporter constructs include the *lacO* operator sequence; constitutive expression is enabled by the absence of LacI repressor in these strains. Promoters were categorized in low (left) or high (right) expression level groups. Illumination conditions were identical within each group, with higher illumination power and longer exposure for the low expression group than for the high expression group (see Material and Methods section). We used identical brightness and contrast settings for displaying fluorescence images obtained for all promoters within each group, hence some fluorescence images displayed here contained saturated pixels (shown in Fig. S1A) due to the marked variation in promoter strengths (measured in C from unsaturated raw images). Scale bar: 2 µm. (C) Distributions of the log_10_ values of mean sfGFP fluorescence intensity (total sfGFP fluorescence signal per cell divided by cell area) normalized by the median value obtained from the P_Biofab_*-sfgfp* population within the corresponding group as in (B). The horizontal dashed line at log_10_ = 0 represents the normalization by the P_Biofab_*-sfgfp* median. On each box, the central line is the median, the edges are the 25th and 75th percentiles, and the whiskers extend to the most extreme nonoutlier datapoints. The outliers are plotted as ‘+’ signs. Representative data from one of two biological replicates. Number of cells analysed (*n*) in the low intensity group: WT (*n* = 6275), P*_lacIq_* (*n =* 2408), P*_nptII_* (*n =* 3328), P*_rhoBb_* (*n =* 1808), P*_lac_* (*n =* 4122), P*_lonBb_* (*n =* 2010), and P_Biofab_ (*n =* 5657); in the high intensity group: WT (*n =* 6240), P_Biofab_ (*n =* 3732), P*_dnaKBb_* (*n =* 3411), P*_tac_* (*n =* 2585), and P*_trc_* (*n =* 3246). Statistics (median, percentiles, min, max, whisker values, and outliers counts) are provided in Table S1. Strain numbers: WT (GL734), P*_lacIq_* (GL2515), P*_nptII_* (GL1845), P*_rhoBb_* (GL1848), P*_lac_* (GL1765), P*_lonBb_*(GL1846), P_Biofab_ (GL1844), P*_dnaKBb_* (GL1847), P*_tac_* (GL1766), and P*_trc_* (GL1767).

*Bdellovibrio bacteriovorus* is an obligate predatory bacterium that invades the periplasm of a range of diderm prey bacteria (Sockett 2009). Once inside the prey cell—now termed a bdelloplast—the predator elongates and undergoes multiple rounds of DNA replication (Makowski et al. 2019, Kaljević et al. 2021) before producing progeny through nonbinary division. Daughter cells, whose number is proportional to prey size (Santin et al. 2023), then escape the bdelloplast and resume hunting (Harding et al. 2020). This distinctive lifecycle (Fig. 1A) entails a nonreplicative attack phase (AP) outside the prey and an intraperiplasmic growth phase (GP), which together imply a complex developmental process. The suite of genes required for coordinating cell cycle transitions with predatory behaviours remains largely unknown (Laloux 2020, Caulton and Lovering 2023, Lai et al. 2023). Genetic approaches to investigate the determinants of the *B. bacteriovorus* cell cycle and prey–predator interactions have typically relied on gene deletion or disruption. However, this strategy can only be applied to nonessential genes, which are fewer in an obligate predator that requires efficient predatory capacity for survival. Multiple studies have bypassed this issue by deleting genes of interest in mutant, prey-independent strains [traditionally qualified as host-independent (HI)]. However, HI strains exhibit various phenotypes and mutations (Cotter and Thomashow 1992, Thomashow and Cotter 1992, Roschanski et al. 2011, Capeness et al. 2013, Rotem et al. 2015), and their growth and division cycle remains incompletely characterized. Gene overexpression (typically from broad host-range replicative plasmids carrying the RSF1010 origin) has also been used to perturb cellular processes (Kaljević et al. 2023), but this approach provides no control over the timing of gene expression. As a result, the development of inducible systems is crucial for studying gene function in *B. bacteriovorus*, particularly for genes that are important or essential for predation and cell cycle control.

Riboswitches have been used to regulate gene expression in *B. bacteriovorus* (Dwidar and Yokobayashi 2017), but these systems generally lack the precise tunability of promoter/repressor systems. Recently, Salgado et al. systematically analysed a set of candidate promoters designed for synthetic biology compatibility and identified the crystal violet-inducible P_JExD_/EliR as a suitable system for gene expression control in *B. bacteriovorus* (Salgado et al. 2024). This system confers tight control over a broad induction range in growth-phase predators without impairing predatory capacities (Salgado et al. 2024). However, fluorescence from crystal violet restricts the use of this promoter in microscopy or spectrometry experiments involving red fluorescent proteins or dyes, due to their overlapping excitation/emission spectra. By contrast, derivatives of the *lac* operon promoter (P*_lac_*) are routinely used in numerous bacterial species to induce gene expression upon addition of isopropyl β-D-1-thiogalactopyranoside (IPTG), which displaces the LacI repressor from the *lacO* operator sequence (Wilson et al. 2007). In *B. bacteriovorus*, however, previous applications of P*_lac_* have yielded either constitutive expression or a lack of IPTG-dependent induction (Flannagan et al. 2004, Roschanski and Strauch 2011, Dwidar and Yokobayashi 2017).

CRISPR interference (CRISPRi) uses a catalytically inactive Cas9 (dCas9) combined with a single guide RNA (sgRNA), which is composed of a 20-bp spacer sequence—complementary to the target sequence—and a Cas9-specific scaffold RNA. The resulting dCas9/sgRNA ribonucleoprotein complex binds DNA and blocks transcription of the targeted gene or operon (Larson et al. 2013, Qi et al. 2013). When combined to an inducible expression system, this approach allows inducible gene knockdown, making it invaluable for studying essential genes and dynamically regulating gene expression. In recent years, the construction of inducible CRISPRi modules has greatly facilitated genetic manipulation in a variety of organisms, including *Borrelia burgdorferi* (Takacs et al. 2020), *Caulobacter crescentus* (Irnov et al. 2017, Guzzo et al. 2020), *Corynebacterium glutamicum* (Cleto et al. 2016), *Pseudomonas aeruginosa* (Tan et al. 2018), *Staphylococcus aureus* (Reed et al. 2024), *Streptococcus pneumoniae* (Liu et al. 2017), and *Vibrio cholerae* (Caro et al. 2019). While the use of CRISPRi has been mentioned in a *B. bacteriovorus* study (Sathyamoorthy et al. 2020), the system was not inducible.

Here, we expand the genetic toolbox available for *B. bacteriovorus*. First, we analyse a range of native, exogenous, and synthetic promoters to compare their relative strengths in driving gene expression. We then report the development of an IPTG-inducible gene expression system that functions during both the attack and growth phases of *B. bacteriovorus*. Finally, we describe the construction of an inducible CRISPRi module, which enables targeted gene knockdown and may be used to circumvent challenges imposed by genes important or essential for predation and survival. These advancements lay the groundwork for a more versatile and precise genetic manipulation of *B. bacteriovorus*, thereby offering new opportunities to investigate its unique lifecycle.

## Materials and methods

### Bacterial strains and growth conditions

*Bdellovibrio bacteriovorus* strains were routinely grown in the presence of *E. coli* MG1655 as prey in liquid DNB (diluted nutrient broth) medium supplemented with 2 mM CaCl_2_ and 3 mM MgCl_2_ at 30°C with agitation or in double-layer DNB agar plates in which the top, soft-agar layer contained a lawn of prey, as described previously (Remy et al. 2022). To obtain prey suspensions, *E. coli* cells were grown in LB medium before washing and resuspending in 2-[4-(2-hydroxyethyl)piperazin-1-yl]ethanesulfonic acid solution supplemented with 2 mM calcium chloride as in Remy et al. (2022). *Escherichia coli* strains carrying an antibiotic resistance cassette were used as prey when selecting antibiotics-resistant *B. bacteriovorus*. When appropriate, kanamycin was added to the *B. bacteriovorus* or *E. coli* cultures at a final concentration of 50 µg/ml. For gene expression induction or for gene silencing via induction of *dcas9_Spa_* expression, IPTG was added to the *B. bacteriovorus* culture at the indicated times upon mixing with prey, at the indicated concentrations starting from a 100 mM working stock solution prepared in dH_2_O.

### Construction of strains and plasmids

All strains and plasmids used in this study are provided in Table S2. *Bdellovibrio bacteriovorus* strains are derived from the wild-type HD100 reference strain (Rendulic et al. 2004), which does not encode an endogenous CRISPR system. Construction methods are detailed in Table S3. Primers sequences are provided in Table S4. Briefly, plasmids were assembled using the NEB HiFi DNA assembly method (New England Biolabs) and verified by Sanger sequencing of the insert or Nanopore sequencing of the whole plasmid in the case of pCRISP and pCRISP-I (Eurofins). *Escherichia coli* strains were transformed routinely by chemical transformation. Plasmids were transferred to *B. bacteriovorus* by conjugation using the S17-1 λ*pir E. coli* strain as donor, following the procedure described previously (Kaljević et al. 2023). Replicative plasmids used in *B. bacteriovorus* carried the low-copy RSF1010 replicon. Transconjugants were selected upon growth with the relevant antibiotic (using *E. coli* MG1655 strains carrying the same antibiotics-resistance cassette as prey) and validated by PCR with primers annealing on the plasmid region flanking the insert. Chromosomal replacements in *B. bacteriovorus* were obtained via a two-step recombination strategy selecting first the integration of a pK18*mobsacB*-derived suicide vector (carrying the Kan^R^ cassette conferring resistance to kanamycin and *sacB* gene conferring sensitivity to sucrose), then plasmid excision by counter-selection of the *sacB* marker in the presence of sucrose. Plasmid loss was further confirmed by verifying kanamycin sensitivity. Clones were picked from single plaques and tested or stored after minimum two rounds of isolation on double-layer DNB agar plates (Remy et al. 2022). The second cross-over yields either the wild-type or the mutant genotype, determined by PCR using primers that hybridize specifically on the chromosome, upstream and downstream the modified locus. Mutants were verified by sequencing of the modified locus. The CRISPRi starter strain GL2000 was also verified by whole-genome Nanopore sequencing (Eurofins).

### Promoters used for IPTG-inducible gene expression and CRISPRi modules

The choice of P*_tac_* for IPTG-inducible gene expression was determined empirically after testing various constructs. P*_lac_*-driven expression (in the absence of the LacI repressor) was relatively weak (see Fig. 1C), and the presence of *lacI* in our initial P*_lac_*-based constructs did not yield obvious IPTG-dependent expression. In contrast, whereas the P*_trc_* promoter showed strong expression (Fig. 1C), it was leakier than P*_tac_* under noninduced conditions. Based on these initial observations, we selected P*_tac_* as the optimal compromise between induction strength and tightness.

To express *lacI* constitutively, we initially used the P*_nptII_* promoter, guided by its prior use in *B. bacteriovorus*, its moderate strength in our tests, and the aim of avoiding excessive LacI levels, which could require higher IPTG concentrations for induction. However, during CRISPRi optimization, we found that stronger repression of *dcas9_Spa_* was needed under noninduced conditions. This led us to use the stronger *dnaK_Bb_* promoter (Fig. 1C) to drive *lacI* expression in the chromosomally encoded CRISPRi module.

### Design of sgRNA for CRISPRi

The use of the *S. pasteurianus* dCas9 in this study was inspired by the implementation of CRISPRi in *C. crescentus* (Guzzo et al. 2020). A sgRNA is composed, from 5′-end to 3′-end, of a 20-bp spacer and a dCas9 handle sequence (Qi et al. 2013). In our hands, a spacer sequence complementary to the nontemplate strand and targeting either the -10/-35 region of the promoter, the region between the transcription start site and the start codon, or—when no suitable site exists upstream—an area close to the start codon within the coding sequence, yielded the strongest repression, consistent with previous reports (Qi et al. 2013, Guzzo et al. 2020). The 20-bp target sequence (also known as protospacer) must be located immediately upstream (when considered with a 5′–3′ orientation on the template strand) of a protospacer-adjacent motif (PAM) appropriate for *S. pasteurianus* dCas9 (Ran et al. 2015, Tan et al. 2018). Here, we used the consensus PAM sequence NNGTGA (Ran et al. 2015), as this motif provided the most effective repression by dCas9*_Spa_*-mediated CRISPRi in *P. aeruginosa* compared to alternative PAM sites (Tan et al. 2018), which were not tested systematically in this study. Using the SnapGene software (www.snapgene.com), we found 35 734 occurrences of the NNGTGA sequence in the *B. bacteriovorus* HD100 reference genome, representing an average of 9.45 PAM sites per kilobase. Promoters were identified using online tools such as BPROM (Solovyev and Salamov 2011). PAM sequences and corresponding target sequences were identified in the region of interest in the *B. bacteriovorus* HD100 genome using the CHOPCHOP tool (Labun et al. 2019). Construction methods for the GL2000 CRISPRi starter strain, in which *dcas9_Spa_* is expressed under the control of an IPTG-inducible P*_tac_* on the *B. bacteriovorus* chromosome, and the pCRISP and pCRISP-I replicative plasmids constitutively expressing the sgRNA, are detailed in Table S3.

### Killing assays and kinetics fluorescence measurements in microplates

Killing assays and kinetics fluorescence measurements were performed essentially as in (Remy et al. 2022). Briefly, fresh AP *B. bacteriovorus* cells from an overnight predatory culture were filtered through 1.2 and 0.8 µm syringe filters to eliminate residual prey and bdelloplasts, and the predator count (in PFU/ml) was estimated upon a SYBR Green labelling assay described in (Remy et al. 2022). The equivalent of 2.4 × 10^8^ PFU of filtered AP suspensions were added per well containing DNB and *E. coli* MG1655 prey at final OD_600_ = 1 (measured in a 1-cm spectrometer cuvette), in a black 96-well plate with transparent bottom. Absorbance at 600 nm and tdTomato fluorescence (excitation 548 nm, emission 586 nm) were recorded in a Synergy H1 m plate reader (Biotek) every 20 min, with continuous double orbital shaking and temperature set to 30°C. Each condition was tested in technical triplicates (different wells in the same plate), and each experiment was reproduced at least twice with fresh cultures (biological replicates). The CuRveR package (Remy et al. 2022) was used to plot the data, to fit curves to a Richard equation and to extract *rmax* values, as previously described.

### Live-cell imaging by phase contrast and fluorescence microscopy

For time-course imaging of the predatory GP, fresh overnight AP *B. bacteriovorus* cells were filtered and 120 µl were added to a suspension of *E. coli* (final OD = 0.8) freshly prepared from an exponentially growing culture, in a final volume of 600 µl, which ensured simultaneous infection of most *E. coli* cells by one *B. bacteriovorus* cell. The mix was incubated at 30°C with shaking and samples were imaged at the indicated time-points after spotting cells on 1.2% agarose pads prepared with DNB medium. For snapshots of AP cells, fresh overnight *B. bacteriovorus* were spotted directly on agarose pads for imaging.

**Figure 2.**
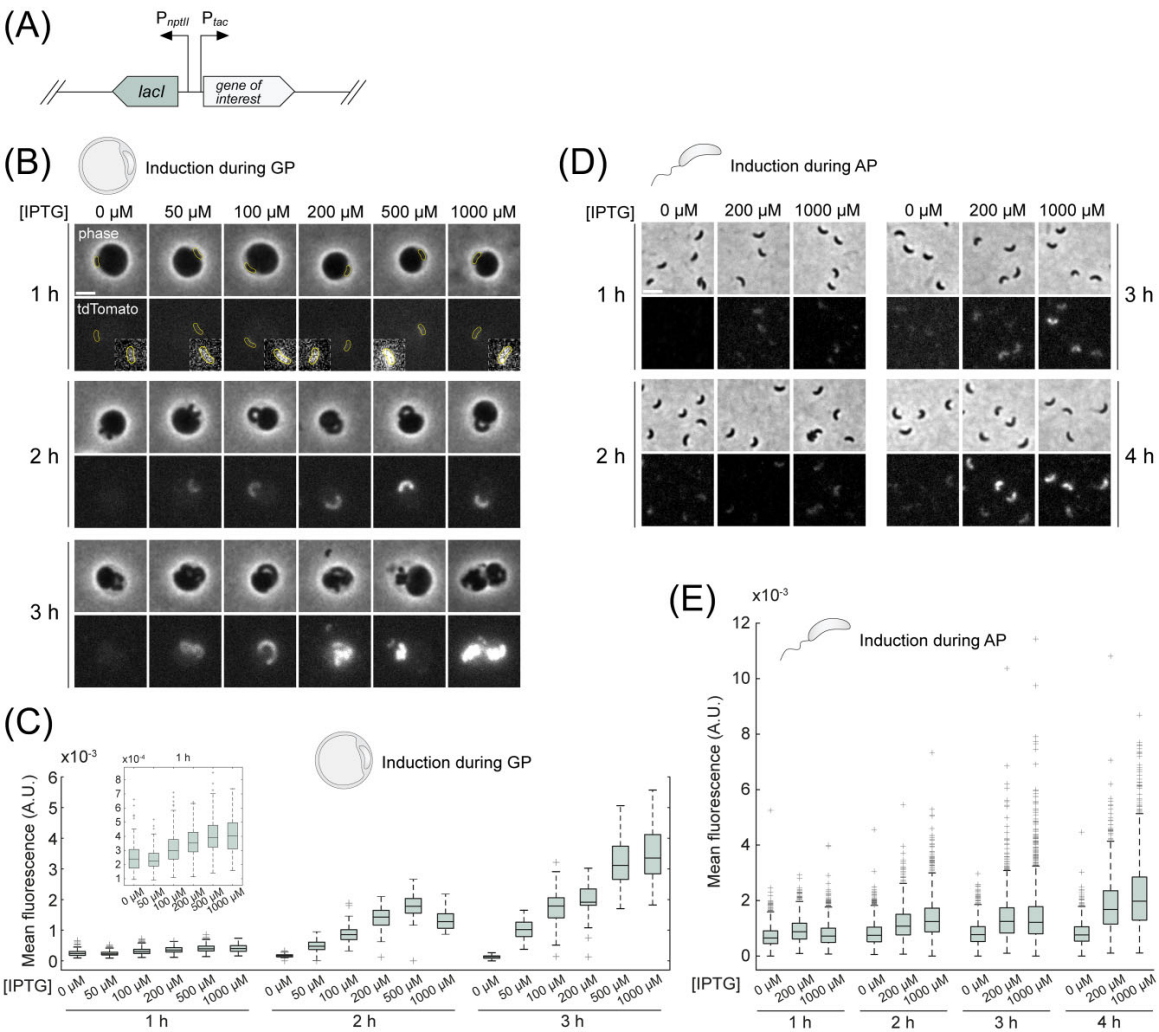
An IPTG-inducible expression system that functions during both the attack and GPs of the *B. bacteriovorus* lifecycle. (A) Schematics of the genetic construct including the *lacI* gene encoding the LacI repressor under control of the constitutive P*_nptII_* promoter, and the P*_tac_* promoter (followed by the *lacO* operator sequence for LacI binding, not depicted) controlling the gene of interest. (B) Representative phase contrast and tdTomato fluorescence images of bdelloplasts, captured at the indicated times after adding *E. coli* prey and various IPTG concentrations (shown on top) to the *B. bacteriovorus* GL2234 strain carrying the chromosomally encoded P*_nptII_-lacI* P*_tac_-tdTomato* construct. *B. bacteriovorus* cell outlines obtained with Oufti are shown for the 1-h condition to facilitate visualization of the low signal. Insets show the same cells as in the main regions of interest, zoomed and displayed with stronger brightness and contrast (identical for all insets) to visualize the IPTG-dependent signal increase. Scale bar: 2 µm. (C) Distributions of mean tdTomato fluorescence intensity (total fluorescence signal per predator cell divided by cell area) from cells imaged as described in (B), in arbitrary units (A.U.). Inset: values obtained for the 1-h induction condition represented using a lower Y-axis scaling to facilitate visualization of the overall signal increase with increasing IPTG concentrations. Boxplot features represent statistical values of the distributions as in Fig. 1C. Representative data from one of two biological replicates. Number of cells analysed (*n*), 1 h induction: 0 µM (*n =* 130), 50 µM (*n =* 146), 100 µM (*n =* 248), 200 µM (*n =* 265), 500 µM (*n =* 284), 1000 µM (*n =* 286); 2 h induction: 0 µM (*n =* 103), 50 µM (*n =* 85), 100 µM (*n =* 92), 200 µM (*n =* 53), 500 µM (*n =* 47), 1000 µM (*n =* 33); 3 h induction: 0 µM (*n =* 27), 50 µM (*n =* 43), 100 µM (*n =* 56), 200 µM (*n =* 27), 500 µM (*n =* 44), and 1000 µM (*n =* 40). Statistics (median, percentiles, min, max, whisker values, and outliers counts) are provided in Table S1. (D) Representative phase contrast and tdTomato fluorescence images of AP *B. bacteriovorus* cells carrying the chromosomally encoded P*_nptII_-lacI* P*_tac_-tdTomato* construct (strain GL2234), captured at the indicated times after adding various IPTG concentrations (shown on top). Scale bar: 2 µm. (E) Distributions of mean tdTomato fluorescence intensity (total fluorescence signal per predator cell divided by cell area) from cells imaged as described in (D), in A.U.. Boxplot features represent statistical values of the distributions as in Figs. 1C and 2C. Four outliers with mean fluorescence values higher than 0.0012 are not shown for clarity. Representative data from one of three biological replicates. Number of cells analysed (*n*), 1-h induction: 0 µM (*n =* 1620), 200 µM (*n =* 1145), 1000 µM (*n =* 2151); 2 h induction: 0 µM (*n =* 2228), 200 µM (*n =* 656), 1000 µM (*n =* 1123); 3 h induction: 0 µM (*n =* 1191), 200 µM (*n =* 993), 1000 µM (*n =* 1955); 4 h induction: 0 µM (*n =* 1136), 200 µM (*n =* 702), and 1000 µM (*n =* 947). Statistics (median, percentiles, min, max, whisker values, and outliers counts) are provided in Table S1.

Phase contrast and fluorescence images were acquired on a Nikon Ti2-E inverted epifluorescence microscope (Nikon) equipped with a CFI Plan Apochromat λDM 100x NA = 1.45 Ph3 oil objective (Nikon), a Sola SEII FISH led illuminator (Lumencor), a Prime95b sCMOS 25-mm field-of-view camera (Photometrics), and filter-cubes for mCherry (32 mm, excitation 562/40, dichroic 593, emission 640/75; Nikon) and GFP (32 mm, excitation 466/40, dichroic 495, emission 525/50; Nikon). Multidimensional acquisition was achieved with the NIS-Ar software (Nikon). Using the 1.5X built-in zoom lens of the microscope, pixel size was 0.074 µm. Identical illumination conditions were used to capture images of the compared strains and conditions within a single experiment. Fluorescence illumination conditions (Lumencor SOLA FISH II Led power and exposure time) were the following, for Fig. 1B and C: 100% 1000 ms (low expression level) and 50% 50 ms (high expression level); Fig. 2B and C: 15% 100 ms; Fig. 2D–E: 50% 200 ms. Note that in Fig. 1B and C, fluorophores likely had different photobleaching efficiencies due to the different light power and exposure times used for the two sets of promoters. This is why we included a promoter as a common reference (P_Biofab_) in both sets: our normalization of fluorescence intensity values in each dataset by the median value obtained from P_Biofab_ in the corresponding dataset allows comparison of promoter strengths relative to this reference promoter, under each of the two illumination conditions.

### Image processing

Microscopy images were processed with Fiji (Schindelin et al. 2012) to crop regions of interest, adjust brightness and contrast, and add scale bars. Image scale, brightness, and contrast were kept identical for all images within each figure panel, unless otherwise stated. Saturated pixels in Fig. S1A were displayed using the HiLo lookup table in Fiji. The Fiji ‘smooth’ denoising function was applied on phase contrast images shown in Fig. 2B. Figures were assembled using Adobe Illustrator (Adobe Inc.).

### Image analysis

**Figure 3.**
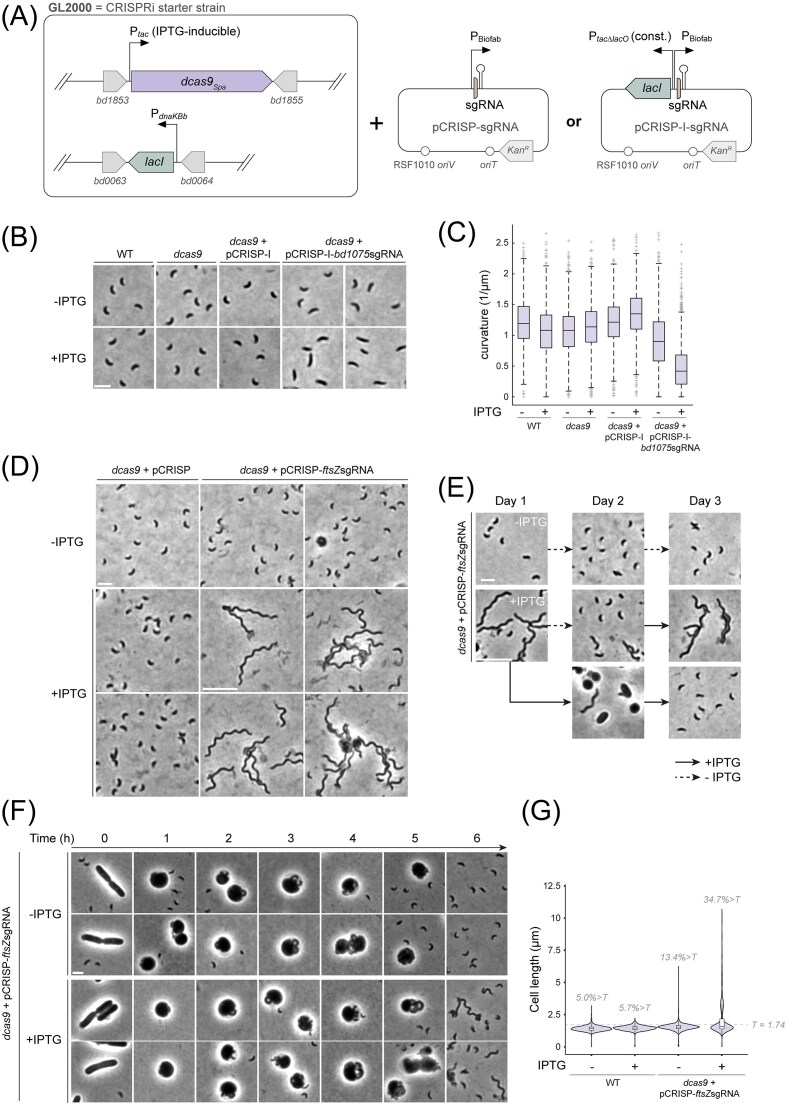
A CRISPRi module for inducible targeted gene silencing in *B. bacteriovorus*. (A) Schematics of the CRISPRi module in *B. bacteriovorus*. In the CRISPRi starter strain GL2000, the gene encoding *S. pasteurianus* dCas9 (*dcas9_Spa_*) is expressed under the control of an IPTG-inducible P*_tac_* promoter at the *bd1853–bd1855* intergenic locus, and *lacI* is expressed constitutively from the *B. bacteriovorus* P*_dnaK_* promoter at the *bd0063–bd0064* intergenic locus. Replicative vectors based on the pSEVA251 backbone allow the P_Biofab_*-*driven constitutive expression of the sgRNA targeting the gene of interest (see Material and Methods section), with (pCRISP-I) or without (pCRISP) an additional copy of *lacI* expressed from the constitutive P*_tac∆lacO_* promoter (i.e. P*_tac_* without a *lacO* operator sequence, therefore not repressed by LacI). (B) Representative phase contrast images of the indicated *B. bacteriovorus* strains carrying or not the gene encoding dCas9_Spa_, the pCRISP-I vector, and the sgRNA targeting the curvature gene *bd1075*. AP cells were imaged after an overnight predatory culture with or without 200 µM IPTG. Strains from left to right: WT (HD100), GL2000, GL2640, and GL2526. Cells carrying the *bd1075* sgRNA (strain GL2526) and grown in the presence of IPTG display a straighter morphology compared to controls without IPTG induction or lacking sgRNA. (C) Distributions of curvature quantified from cells imaged as in (B). Boxplot features represent statistical values of the distributions as in Figs. 1C and 2C and E. Representative data out of two biological replicates. Strains from left to right and number of cells (*n*) analysed (uninduced, induced): WT (*n =* 756, *n =* 833), GL2000 (*n =* 831, *n =* 1058), GL2640 (*n =* 551, *n =* 550), and GL2526 (*n =* 1212, *n =* 1697). Statistics (median, percentiles, min, max, whisker values, and outliers counts) are provided in Table S1. (D) Representative phase contrast images of the indicated *B. bacteriovorus* strains carrying the gene encoding dCas9_Spa_ and the pCRISP vector with (strain GL2499) or without (strain GL2639) the sgRNA targeting *ftsZ*. AP cells were imaged after an overnight predatory culture with or without 200 µM IPTG. Cells carrying the *ftsZ* sgRNA (GL2499) grown in the presence of IPTG are filamentous, indicating a cell division defect. Scale bars: 2 µm (top left) and 5 µm (middle); all regions of interest were cropped at the same dimensions and scaled identically. (E) Reversibility of CRISPRi-mediated depletion and additional support for *ftsZ* essentiality. The *B. bacteriovorus* GL2499 strain (carrying *dcas9_Spa_* and the pCRISP vector with the sgRNA targeting *ftsZ*) was grown overnight in the presence of *E. coli* MG1655 prey, in the absence (top) or presence (bottom) of IPTG (Day 1). Cells were then subcultured twice, with or without IPTG (plain and dashed arrows, respectively)*. Top row*: negative control without IPTG; all cells display wild-type morphology. *Middle row*: FtsZ-depleted cells (Day 1) mostly recover wild-type cell length when subcultured in absence of inducer (Day 2), and the cell division defect is recapitulated upon reintroduction of the inducer (Day 3). *Bottom row*: FtsZ-depleted cells (Day 1) mostly fail to proliferate and do not elongate further when subcultured in presence of inducer, and many bdelloplasts and uninfected prey are observed (Day 2); additional subculturing with IPTG mostly selects for predators with wild-type morphology—likely suppressor mutants inactivating the CRISPRi system (Day 3). Representative phase contrast images are shown. Scale bars: 2 µm (top) and 5 µm (bottom); all images were scaled identically. (F) Representative phase contrast images of the *B. bacteriovorus* GL2499 strain carrying the gene encoding dCas9_Spa_ and the pCRISP vector with the sgRNA targeting *ftsZ*. AP predators from an overnight predatory culture in absence of IPTG were mixed with *E. coli* MG1655 prey with or without 200 µM IPTG (time 0). Images of culture samples were taken in time-course as indicated. Newborn predators from the induced culture are elongated, indicative of a cell division defect within one generation. Scale bar: 2 µm. (G) Violin plots showing the distribution of *B. bacteriovorus* cell lengths measured from phase contrast images of newborn predators obtained in the experiment described in (F). Boxplots represent statistical values as in Figs. 1C, 2C and E, and 3C (see values in Table S1). For each condition, the fraction of cells with a cell length higher than a threshold value T (1.74, corresponding to the 95^th^ percentile of the cell lengths values for the WT–IPTG condition, represented by the dashed line) is indicated on top of the corresponding violin plot. Strains, mean cell length ± standard deviation (SD), coefficient of variation (CV), and number of cells analysed (*n*) without and with 200 µM IPTG, respectively: WT HD100 (mean ± SD = 1.42 ± 0.21, CV = 0.15, *n =* 1675; mean ± SD = 1.45 ± 0.19, CV = 0.13, *n =* 1015); GL2499 (mean ± SD = 1.53 ± 0.27, CV = 0.18, *n =* 2056; mean ± SD = 2.12 ± 1.6, CV = 0.65, *n =* 1981).

Cell outlines of attack-phase *B. bacteriovorus* were obtained in an automated manner from phase contrast microscopy images using the Oufti software (Paintdakhi et al. 2016), followed by manual correction. For Fig. 2C, cell outlines of growth-phase *B. bacteriovorus* inside bdelloplasts were obtained by manual addition in Oufti. This procedure did not result in whole-cell outlining for all elongated and/or twisted predator cells (especially at the 3-h time point) due to the parameters constraints. Hence, fluorescence signal was normalized by cell area (for Figs. 1C and 2C and E) to reliably measure fluorescence increase in individual cells across all timepoints and induction conditions, regardless of the accuracy of cell outlining. Fluorescence signal was added to the cell lists upon background subtraction in Oufti. Intensity values were extracted from Oufti cell lists and normalized by cell area to obtain mean fluorescence intensity values for individual cells using the *HistMeanFluo.m* custom code (Kaljević et al. 2021) in Matlab. Median values, normalization, and log values were obtained in Matlab. Curvature values were computed as the inverse of the radius of curvature in µm, extracted for all cells from Oufti cell lists using the Oufti *getcurvature.m* function (Paintdakhi et al. 2016), and the resulting data (in 1/µm units) were plotted in Matlab. For Fig. 3G, cell length values were extracted from the Oufti cell lists using the *histCellLength.m* custom code (Kaljević et al. 2023) in Matlab (Mathworks) and plotted as violin plots in R (R Studio) using *ggplot2* (Wickham 2016). Boxplot distributions were computed and plotted in Matlab using the *boxplot* function with default parameters for Figs. 1C and E, 2C, and 3C. Datapoints were considered as outliers if they are larger than Q3+1.5*(Q3–Q1) or smaller than Q1–1.5*(Q3–Q1), where Q1 and Q3 are the 25th and 75th percentiles, respectively. Statistical values used in all boxplots are shown in Table S1.

### Quantification and statistical analysis

Specific software and computing of statistical values for quantitative analysis of different experimental datasets are described in the corresponding methods. Number of cells analysed (*n*) per strain for the representative experiments shown in figures are indicated in the corresponding figure legends. All experiments were performed at least twice, using independent, freshly prepared predator cultures for each biological replicate.

## Results

### Relative strengths of a set of promoters in B. bacteriovorus

First, we systematically examined the relative strengths of a set of native, non-native, and synthetic promoters in driving constitutive expression of a fluorescent reporter (superfolder green fluorescent protein, sfGFP) in *B. bacteriovorus*. We used fluorescence microscopy to assess, at the single-cell level, *sfgfp* reporter expression from a set of constitutive promoters carried on a medium-copy replicative plasmid (pSEVA251 with an RSF1010 origin; see Material and Methods section). The tested promoters include native *B. bacteriovorus* promoters active during both AP and GP (P*_rhoBb_*, P*_lonBb_*, and P*_dnaKBb_*) (Lambert et al. 2010, Karunker et al. 2013, Rotem et al. 2015), a synthetic promoter (P_Biofab_) (Mutalik et al. 2013, Mavridou et al. 2016), and non-native promoters previously used in *B. bacteriovorus* (P*_nptII_*) (Mukherjee et al. 2015, Kaljević et al. 2021, 2023) or routinely employed in model organisms (P*_lac_*, P*_tac_*, P*_trc_*, and P*_lacIq_*). AP predators were imaged by phase contrast and fluorescence microscopy (Figs. 1B and S1A), and the mean sfGFP fluorescence intensity (i.e. total fluorescence per cell normalized by cell area) was monitored for individual cells (Fig. 1C). To accommodate various promoter strengths, we used two different illumination settings. Based on an initial assessment of reporter fluorescence obtained under both conditions, we grouped promoters into low- and high-intensity categories (Fig. 1C, left and right, respectively). This approach allowed us to avoid signal saturation for highly expressing promoters while maintaining sufficient sensitivity for weaker ones. We included a common reference promoter (P_Biofab_) in each group for normalization, enabling comparison of relative promoter strengths across both illumination conditions. Cells carrying the P*_lacIq_*-*sfgfp* construct displayed fluorescence levels comparable to the background signal measured in wild-type strain, indicating that the P*_lacIq_* promoter is likely inactive in *B. bacteriovorus* (Fig. 1C, left). The distributions of signal intensities, normalized by the median fluorescence value from P_Biofab_ of the corresponding group, indicate a range of promoter strengths conferring fluorescence levels above the wild-type background, with P*_nptII_* and P*_trc_* providing the lowest and highest expression, respectively (Fig. 1C, Table S1). Notably, two of the tested promoters, P*_tac_* and P*_trc_*, showed here the strongest expression levels (Fig. 1C, Table S1), representing new powerful tools for driving gene expression in *B. bacteriovorus*. The observed cell-to-cell heterogeneity in fluorescence signal intensity is likely explained by the fact that the reporter constructs are carried on plasmid (Jahn et al. 2016, Salgado et al. 2024); a chromosomally encoded P_Biofab_-*sfgfp* reporter indeed yielded a more homogenous distribution of fluorescence signal (Fig. S1B). Thus, these data both characterize and expand the set of promoters available for constitutive gene expression in *B. bacteriovorus*.

### Construction of an IPTG-inducible gene expression system in B. bacteriovorus

We next sought to develop an easily amenable system for routinely inducing gene expression in *B. bacteriovorus*. Our observation that P*_lacIq_* is inactive in *B. bacteriovorus* (Fig. 1B and C) might account for the reported lack of IPTG-dependent induction in a study where the *P_lacIq_* promoter was used to drive *lacI* expression (Flannagan et al. 2004). In other cases, the gene encoding the LacI repressor appeared not included in the system, potentially explaining the absence of IPTG-inducible activity (Roschanski and Strauch 2011, Dwidar and Yokobayashi 2017). One study noted that a P*_tac_*-driven gene expression required IPTG in *B. bacteriovorus*, although a detailed characterization of the induction was not provided (Duncan et al. 2019). Thus, we revisited the possibility of using an IPTG-responsive promoter in *B. bacteriovorus*. We constructed a module including (i) the *lacI* gene encoding the LacI repressor, constitutively expressed from the P*_nptII_* promoter, and (ii) the P*_tac_* promoter including the *lacO* operator (Fig. 2A; the choice of these promoters is explained in Materials and Methods section). To test IPTG-dependent expression, we placed *tdtomato* under the control of the P*_tac_* promoter and monitored tdTomato fluorescence per bdelloplast over time after adding a range of IPTG concentrations to a fresh prey–predator mix. Fluorescence signal was below the detection limit in the absence of IPTG and increased in a dose-dependent manner when IPTG was supplied concomitantly with prey (Fig. 2B and C, and Table S1).

None of the tested IPTG concentrations (up to 1 mM) altered predatory capacity, which we assessed by the decrease in culture absorbance (Fig. S2A, Table S1; compare WT and P*_tac_-tdtomato*) and by the increase in fluorescence from a constitutively produced tdTomato (Fig. S2B, left), used as proxies for prey lysis and predator proliferation, respectively (Remy et al. 2022). At the population level, fluorescence from the P*_tac_-tdtomato* construct increased with IPTG concentrations (Fig. S2B, middle); 500 µM and 1 mM IPTG produced similar results. Adding 200 µM IPTG yielded fluorescence comparable to constitutive *tdtomato* expression driven by the P_Biofab_ promoter. With P*_tac_-tdtomato*, however, the signal started at a lower, background level at the time of induction and then rose more steeply (Fig. S2B, right). Because both strains exhibited similar prey killing rates (Table S1, compare GL1462 and GL2234), these observations are consistent with IPTG-induced reporter expression occurring alongside population growth. Importantly, the system also functioned in AP cells (Figs. 2D and E, and S2C), despite the generally reduced transcriptional activity during this stage of the cell cycle (Lambert et al. 2010, Karunker et al. 2013). Collectively, these data demonstrate that IPTG-inducible expression works in *B. bacteriovorus*, substantially enriching the existing genetic toolbox.

### Design of a CRISPRi module for targeted gene knockdown in B. bacteriovorus

We then investigated the possibility of exploiting our IPTG-dependent system to implement inducible, targeted gene silencing through CRISPRi in *B. bacteriovorus*. We assembled a module in which *dcas9_Spa_—*encoding dCas9 derived from *Streptococcus pasteurianus* as in Guzzo et al. (2020)—is placed under the control of the IPTG-inducible P*_tac_* promoter described above. This construct was integrated into the chromosome of the wild-type *B. bacteriovorus* HD100 strain at the *bd1853–bd1855* intergenic locus. We also inserted a construct allowing constitutive P*_dnaKBb_*-driven expression of *lacI* at the *bd0063–bd0064* intergenic locus, resulting in the CRISPRi ‘starter strain’ GL2000 (Fig. 3A; see Materials and Methods section). We verified that the predatory capacity of this strain was not modified compared to the wild-type HD100 strain (Fig. S2A and Table S1). Finally, we constructed a replicative vector, named pCRISP, using pSEVA251 as backbone for the P_Biofab_-driven constitutive expression of a sgRNA targeting a gene of interest thanks to a 20 base-pair spacer sequence. A second version of this vector, pCRISP-I, includes an additional copy of *lacI* driven by the strong constitutive P*_tac∆lacO_* promoter (i.e. P*_tac_* lacking the operator sequence, hence not repressed by LacI). This construct provides a tighter IPTG-dependent control of *dcas9_Spa_* expression, which we found required for optimal CRISPRi depending on the target (see below).

### CRISPRi silencing of bd1075 recapitulates the straight phenotype of the deletion mutant

To validate our CRISPRi design, we first selected the *bd1075* gene, which is involved in *B. bacteriovorus* cell curvature through the asymmetric activity of the encoded L, D-carboxypeptidase on the predator's cell wall (Banks et al. 2022). Deletion of *bd1075* had shown a pronounced straight-rod phenotype (Banks et al. 2022), providing a clear morphological readout for CRISPRi-induced depletion. We designed a sgRNA including a 20-bp spacer targeting the coding sequence of *bd1075* (16 base pairs after the start codon; see Materials and Methods section) and expressed from the pCRISP-I vector in the GL2000 strain. Fresh attack-phase predator cells were incubated overnight in the presence of *E. coli* prey and IPTG, and predator progeny were monitored by phase contrast microscopy (Fig. 3B). Only predator cells expressing the *bd1075*-targeted sgRNA displayed a straight morphology, whereas cells with the control vector (lacking the entire sgRNA) retained wild-type curvature. In the absence of IPTG, all strains appeared curved similarly as the wild-type HD100 strain (Fig. 3B). Single-cell curvature measurements (Fig. 3C and Table S1) are consistent with these observations, although we note that the noninduced GL2526 strain (carrying the *bd1075*-targeted sgRNA) showed overall slightly lower curvature values than the control strains, likely reflecting incomplete repression of *dcas9_Spa_* in absence of IPTG. Nevertheless, these values remained higher than in the induced condition. Altogether, these results demonstrate that CRISPRi can be induced in *B. bacteriovorus* to knock down a target gene, recapitulating its knockout phenotype.

### FtsZ depletion through CRISPRi leads to predator cell filamentation

Finally, we extended our CRISPRi validation by targeting an essential gene whose silencing should generate a readily observable, strong phenotype. The tubulin homologue FtsZ, critical for bacterial cell division and essential in most species (Cameron and Margolin 2024), was an attractive target for this purpose. In *B. bacteriovorus*, mNeonGreen-tagged FtsZ formed foci at positions consistent with division sites (Pląskowska et al. 2023). Our repeated attempts to delete *ftsZ* in *B. bacteriovorus* invariably yielded only wild-type genotype after the second recombination event (see Methods, *n =* 2; 100% of 138 clones screened by PCR were wild-type), showing that *ftsZ* is essential for *B. bacteriovorus* survival. We therefore expressed an *ftsZ-*targeting sgRNA from the pCRISP vector in the GL2000 strain and assessed cell morphology following overnight predation in the presence of IPTG. Most cells were filamentous (Fig. 3D), consistent with a cell division defect and demonstrating that CRISPRi can reveal depletion phenotypes for essential genes in *B. bacteriovorus*. Filamentation markedly decreased when these cells were subcultured in the absence of IPTG, and reappeared when IPTG was reintroduced, indicating the reversibility of CRISPRi-mediated depletion (Fig. 3E, middle row). However, upon continued subculturing of overnight FtsZ-depleted cells with IPTG, most cells reverted to a wild-type morphology (Fig. 3E, bottom row)—presumably due to suppressor mutations inactivating *dcas9_Spa_* and/or sgRNA expression—providing additional support for *ftsZ* essentiality. Notably, the impact of FtsZ-depletion was already detectable within the first generation of a synchronized predation cycle, as >30% of cells escaped the bdelloplast in an elongated form, when IPTG was added at the same time as prey bacteria (Fig. 3F and G). Altogether, our findings provide a proof-of-principle for fast, efficient, and reversible gene depletion in *B. bacteriovorus*—including for essential genes—using IPTG-inducible CRISPRi.

## Discussion

The recent expansion of genetic toolboxes, notably through the design of CRISPRi modules, across a wide range of bacterial species has significantly broadened opportunities for molecular and cellular research beyond well-established model organisms. Our work contributes to these efforts by introducing new genetic tools for *B. bacteriovorus*, an obligate predator whose unique lifecycle challenges standard genetic approaches.

By enriching a range of constitutive promoters and establishing an IPTG-inducible system that functions in both attack and growth phases, our study enables the control of gene expression across the entire predatory cycle of *B. bacteriovorus*. Combined with the possibility to synchronize prey invasion, this flexibility is particularly valuable for probing processes active during specific cell cycle stages. For example, initiating gene expression or silencing at the end of the GP may allow the investigation of processes that manifest during the subsequent AP.

The inducible CRISPRi module presented here further broadens the scope for functional studies in *B. bacteriovorus*, especially for genes that cannot be deleted without compromising predatory efficiency and survival. We observed that *dcas9* expression could exhibit some leakiness, underscoring the importance of tuning the regulatory circuit on a case-by-case basis to reduce background effects and enhance depletion, for instance by adjusting *lacI* copy number or expression level, or testing several target sequences.

While our CRISPRi system constitutes an invaluable tool for functional analyses in *B. bacteriovorus*, possible drawbacks must be considered depending on the genomic environment of the targeted gene. Indeed, CRISPRi targeting of a gene within an operon is expected to silence downstream genes as well, calling for additional controls—such as complementation assays—when the genetic basis of an observed phenotype must be unambiguously identified. Alternatively, knocking down a single gene within an operon may be achieved by adding a copy of downstream genes—ideally under control of their native promoter—at a different location on the chromosome. The gene *bd1075* targeted in our study is not part of an operon, and the downstream gene is on the other strand, precluding polar effects of CRISPRi-mediated silencing in this case. On the other hand, FtsZ is encoded in a predicted operon (*ftsA–ftsZ–lpxH–Bd3187*; BioCyc ID TU2753-1553). However, genes downstream of *ftsZ* are likely not directly involved in the massive cell division defect obtained by CRISPRi: *lpxH* is involved in LPS biosynthesis and *bd3187* encodes a hypothetical protein. Our target sequence was located 374 bp downstream of the *ftsZ* start codon, hence not affecting the transcription of *ftsA* situated upstream of *ftsZ*.

We expect the rapid, inducible, and reversible knockdown of essential genes in *B. bacteriovorus* to reveal previously inaccessible aspects of both its noncanonical cell cycle and predatory lifestyle. Future applications may include dissecting how *B. bacteriovorus* coordinates its developmental transitions—for instance, our data on FtsZ-depletion indicate that cell division is not a prerequisite for prey exit—and exploring potential biotechnological uses of predatory bacteria. Overall, the new tools presented here allow for more precise interrogation of gene function in this intriguing model of bacterial predation.

## Supplementary Material

uqaf021_Supplemental_Files
