# Supplementary material for: Expanding the genetic toolbox of the obligate predatory bacterium Bdellovibrio bacteriovorus with inducible gene expression and CRISPR interference
Source: Microlife. 2025 Sep 1;6:uqaf021. doi: 10.1093/femsml/uqaf021 (PMC12448681; doi:10.1093/femsml/uqaf021)
Supplement: uqaf021_Supplemental_Files [file uqaf021_supplemental_files.zip › R1_SupplLegends_de Pierpont et al.docx]

**Figure S1. Supporting data related to Figure 1B-C. (A)** Saturated pixels in the displayed regions of interests of Fig. 1B. Shown are regions of interest from Fig. 1B in which pixels are saturated (indicated in red), as expected from the marked differences in strengths across the tested promoters (see quantification in Fig. 1C) and the identical brightness and contrast settings used for displaying all regions of interest within each category. Note that we determined the appropriate illumination conditions for each category such that the raw fluorescence images used for analysis in Fig. 1C were not saturated. **(B)** Distributions of mean fluorescence intensity (total sfGFP fluorescence signal per cell divided by cell area) in populations of attack-phase *B. bacteriovorus* carrying the P_Biofab_-*sfgfp* reporter construct at a chromosomal locus (strain GL1212) or on a replicative pSEVA251 plasmid (strain GL1844). Plasmid-encoded reporter is associated with slightly higher median fluorescence and more heterogenous distribution compared to the chromosomally encoded reporter. On each box, the central line is the median (0.64x10^-3^ and 1.04x10^-3^ for GL1212 and GL1844, respectively), the edges are the 25th and 75th percentiles, and the whiskers extend to the most extreme non-outlier datapoints. The outliers are plotted as “+” signs. Number of cells analysed (n): n = 982 (GL1212), n = 1082 (GL1844). Representative data from one of two biological replicates. Statistics (median, percentiles, min, max, whisker values, and outliers counts) are provided in **Table S1**.

**Figure S2. Predatory efficiency in the presence of IPTG, and IPTG-dependent induction of reporter gene expression during the attack and growth phases*.* (A)** Kinetics of absorbance (optical density at 600 nm, OD_600_) for the WT HD100 *B. bacteriovorus* strain, the GL2234 strain carrying the chromosomally encoded P*_nptII_-lacI Ptac-tdTomato* construct, and the CRISPRi starter strain GL2000 (from left to right), monitored at the population-level in micro-wells upon mixing with prey and IPTG at the indicated concentrations. Prey alone: negative control without *B. bacteriovorus*. Curves obtained for all three strains and the prey alone condition using 200 µM IPTG are compiled on the right for comparison. Lines represent the fit calculated with CuRveR from technical triplicates; shaded areas represent standard deviations. Values of OD_600_ *rmax* computed with CuRveR and corresponding to the prey killing rate, are provided for all replicates in **Table S1**. **(B)** Kinetics of tdTomato fluorescence for the *B. bacteriovorus* GL1462 strain constitutively producing tdTomato via P_Biofab_-driven expression on the chromosome (left) and the GL2234 strain carrying the chromosomally encoded P*_nptII_-lacI* P*_tac_-tdtomato* construct (middle), compared to the wild-type HD100 strain as negative control of fluorescence (right), monitored at the population-level in micro-wells upon mixing with prey and adding IPTG at the indicated concentrations. Lines represent the fit calculated with CuRveR from technical triplicates; shaded areas represent standard deviations; A.U.: arbitrary units. There was no obvious difference in prey killing rate computed for GL1462 compared to GL2234 and WT strains (see **Table S1**), supporting the idea that the lower starting fluorescence values and steeper increase for GL2234 in presence of 200 µM IPTG is due to the induction of *tdtomato* gene expression during predator growth, in contrast with constitutive tdTomato production in GL1462. **(C)** Kinetics monitoring of tdTomato fluorescence in suspensions of attack phase *B. bacteriovorus* HD100 (WT) or *B. bacteriovorus* cells carrying the chromosomally encoded P*_nptII_-lacI* P*_tac_-tdTomato* construct (strain GL2234). Fresh attack phase cells from overnight predatory cultures were filtered to discard remaining *E. coli* and bdelloplasts before addition of IPTG at the indicated concentrations and incubation in a 96-well plate for measurements. DNB: control medium without *B. bacteriovorus*; A.U.: arbitrary units. Lines represent the fit calculated with CuRveR from technical triplicates; shaded areas represent standard deviations. **(A-C)** Data are from one representative experiment out of three biological replicates.
