# Supplementary material for: Expanding the genetic toolbox of the obligate predatory bacterium Bdellovibrio bacteriovorus with inducible gene expression and CRISPR interference
Source: Microlife. 2025 Sep 1;6:uqaf021. doi: 10.1093/femsml/uqaf021 (PMC12448681; doi:10.1093/femsml/uqaf021)
Supplement: uqaf021_Supplemental_Files [file uqaf021_supplemental_files.zip › R1_Table S2-S3-S4_de Pierpont et al_plaintext.docx]

**Table S2 – Bacterial strains used in this study**

| **Strains** | **Description** | **Resistance** | **Source** |
| --- | --- | --- | --- |
| ***Bdellovibrio bacteriovorus*** | | | |
| GL734 | Wild-type *B. bacteriovorus* HD100 | - | Lab collection (Kind gift from R.E. Sockett, U. Nottingham, UK); (Rendulic *et al.* 2004) |
| GL1212 | HD100 *bd0063-bd0064*::P_Biofab_*-sfgfp* | - | This study |
| GL1462 | HD100 *bd0063-bd0064*::P_Biofab_*-tdtomato* | - | (Santin *et al.* 2023) |
| GL1765 | HD100 / pSEVA251-P*_lac_-sfgfp* | Kan | This study |
| GL1766 | HD100 / pSEVA251-P*_tac_-sfgfp* | Kan | This study |
| GL1767 | HD100 / pSEVA251-P*_trc_-sfgfp* | Kan | This study |
| GL1844 | HD100 / pSEVA251-P_Biofab_*-sfgfp* (pBG18) | Kan | This study |
| GL1845 | HD100 / pSEVA251-P*_nptII_-sfgfp* | Kan | This study |
| GL1846 | HD100 / pSEVA251-P*_lonBb_-sfgfp* | Kan | This study |
| GL1847 | HD100 / pSEVA251-P*_dnaKBb_-sfgfp* | Kan | This study |
| GL1848 | HD100 / pSEVA251-P*_rhoBb_-sfgfp* | Kan | This study |
| GL2000 | HD100 *bd0063-bd0064*::P*_dnaK_-lacI*, *bd1853-bd1855*::P*_tac_-dcas9_Spa_* | - | This study |
| GL2234 | HD100 *bd0063-bd0064*::P*_nptII_-lacI-*P*_tac_-tdtomato* | - | This study |
| GL2415 | HD100 *bd0063-bd0064*::P*_dnaK_-lacI* | - | This study |
| GL2499 | HD100 *bd0063-bd0064*::P*_dnaK_-lacI*, *bd1853-bd1855*::P*_tac_-dcas9_Spa_* / pCRISP-*ftsZ*sgRNA | Kan | This study |
| GL2515 | HD100 / pSEVA251-P*_lacIq_-sfgfp* | Kan | This study |
| GL2526 | HD100 *bd0063-bd0064*::P*_dnaK_-lacI*, *bd1853-bd1855*::P*_tac_-dcas9_Spa_* / pCRISP-I*-bd1075*sgRNA | Kan | This study |
| GL2639 | HD100 *bd0063-bd0064*::P*_dnaK_-lacI*, *bd1853-bd1855*::P*_tac_-dcas9_Spa_* / pSEVA251-P_Biofab_-Terminator (empty pCRISP) | Kan | This study |
| GL2640 | HD100 *bd0063-bd0064*::P*_dnaK_-lacI*, *bd1853-bd1855*::P*_tac_-dcas9_Spa_* / pSEVA251-P*_tac_-lacI-*P_Biofab_-Terminator (empty pCRISP-I) | Kan | This study |
| ***Escherichia coli*** | | | |
| GL574 | S17-1 λ*pir*, used as donor strain for conjugation | Strep | Lab collection |
| GL655 | Wild-type *E. coli* MG1655, used as prey | - | Lab collection |
| GL818 | MG1655 / pSEVA281∆*oriT*, used as Kan^R^ prey | Kan | This study |
| GL669 | TOP10 / pK18mobsacB | Kan | Lab collection |
| GL728 | DH5⍺ / pBG18 (pSEVA251-P_Biofab_*-sfgfp*) | Kan | Kind gift from S. Bigot & C. Lesterlin (CNRS Lyon, FR) |
| GL735 | S17-1 λ*pir* / pBG18 (pSEVA251-P_Biofab_*-sfgfp*) | Strep Kan | This study |
| GL972 | S17-1 λ*pir* / pK18mobsacB- *bd0063*-P_Biofab_-*sfgfp-bd0064* | Strep Kan | This study |
| GL1537 | TOP10 / pBG18_FlippedBioFab_RBS | Kan | This study |
| GL1729 | S17-1 λ*pir* / pSEVA251-P*_lac_-sfgfp* | Strep Kan | This study |
| GL1730 | S17-1 λ*pir* / pSEVA251-P*_tac_-sfgfp* | Strep Kan | This study |
| GL1731 | S17-1 λ*pir* / pSEVA251-P*_trc_-sfgfp* | Strep Kan | This study |
| GL1829 | S17-1 λ*pir* / pSEVA251-P*_nptII_-sfgfp* | Strep Kan | This study |
| GL1830 | S17-1 λ*pir* / pSEVA251-P*_lacIq_-sfgfp* | Strep Kan | This study |
| GL1831 | S17-1 λ*pir* / pSEVA251-P*_lonBb_-sfgfp* | Strep Kan | This study |
| GL1832 | S17-1 λ*pir* / pSEVA251-P*_dnaKBb_-sfgfp* | Strep Kan | This study |
| GL1833 | S17-1 λ*pir* / pSEVA251-P*_rhoBb_-sfgfp* | Strep Kan | This study |
| GL2247 | S17-1 λ*pir* / pK18mobsacB-*bd0063*-P*_nptII_-lacI-*P*_tac_*-*tdtomato-bd0064* | Strep Kan | This study |
| GL2410 | S17-1 λ*pir* / pK18mobsacB- *bd0063*-P*_dnaKBb_*-*lacI*-*bd0064* | Strep Kan | This study |
| GL2472 | S17-1 λ*pir* / pCRISP-*ftsZ*sgRNA | Strep Kan | This study |
| GL2473 | S17-1 λ*pir* / pK18mobsacB-*bd1853*-P*_dnaKBb_*-*lacI*-P*_tac_-dcas9_Spa_*-*bd1855* | Strep Kan | This study |
| GL2524 | S17-1 λ*pir* / pCRISP-I*-bd1075*sgRNA | Strep Kan | This study |
| GL2636 | S17-1 λ*pir* / pCRISP (pSEVA251-P_Biofab_-Terminator) | Strep Kan | This study |
| GL2637 | S17-1 λ*pir* / pCRISP-I (pSEVA251-P*_tac_-lacI-*P_Biofab_-Terminator) | Strep Kan | This study |

Abbreviations: Strep: streptomycin; Kan: kanamycin

**Table S3 – Strains and plasmids construction**

| **Strains** | **Construction** |
| --- | --- |
| GL1212 | Mating GL734 x GL972, followed by 2-step recombination for chromosomal integration (selection of Kan^R^ clones) and excision of the vector (selection of Suc^R^ clones, verification of Kan^S^) |
| GL1765 | Mating GL734 x GL1729, selection of Kan^R^ clones |
| GL1766 | Mating GL734 x GL1730, selection of Kan^R^ clones |
| GL1767 | Mating GL734 x GL1731, selection of Kan^R^ clones |
| GL1844 | Mating GL734 x GL735, selection of Kan^R^ clones |
| GL1845 | Mating GL734 x GL1829, selection of Kan^R^ clones |
| GL1846 | Mating GL734 x GL1831, selection of Kan^R^ clones |
| GL1847 | Mating GL734 x GL1832, selection of Kan^R^ clones |
| GL1848 | Mating GL734 x GL1833, selection of Kan^R^ clones |
| GL2000 | Mating GL2415 x GL2473, followed by 2-step recombination for chromosomal integration (selection of Kan^R^ clones) and excision of the vector (selection of Suc^R^ clones, verification of Kan^S^) |
| GL2234 | Mating GL734 x GL2247, followed by 2-step recombination for chromosomal integration (selection of Kan^R^ clones) and excision of the vector (selection of Suc^R^ clones, verification of Kan^S^) |
| GL2415 | Mating GL734 x GL2410, followed by 2-step recombination for chromosomal integration (selection of Kan^R^ clones) and excision of the vector (selection of Suc^R^ clones, verification of Kan^S^) |
| GL2499 | Mating GL2000 x GL2472, selection of Kan^R^ clones |
| GL2515 | Mating GL734 x GL1830, selection of Kan^R^ clones |
| GL2526 | Mating GL2000 x GL2524, selection of Kan^R^ clones |
| GL2639 | Mating GL2000 x GL2636, selection of Kan^R^ clones |
| GL2640 | Mating GL2000 x GL2637, selection of Kan^R^ clones |
| **Plasmids** | **Construction** |
| pBG18_FlippedBioFab_RBS | Assembly of the following PCR-amplified fragments: vector amplied from pBG18 (GL728) using primers oGL1351 and oGL1515; P_Biofab_ amplified from pBG18 (GL728) using oGL1516 and oGL1517. The resulting plasmid is a pSEVA251-derived vector with the constitutive synthetic P_Biofab_ promoter flipped in opposite direction from the promoter-less *sfgfp* reporter. |
| pSEVA251-P*_lac_-sfgfp* | Assembly of the following fragments: vector amplified by PCR from pBG18_FlippedBioFab_RBS (GL1537) using primers oGL1518 and oGL1519; P*_lac_* and *lacO* amplified by PCR from pAM238 (Gil and Bouche 1991) using oGL1524 and oGL1525. The resulting plasmid is a pSEVA251-derived vector with the *sfgfp* reporter gene under control of the P*_lac_* promoter. |
| pSEVA251-P*_tac_-sfgfp* | Assembly of the following fragments: vector amplified by PCR from pBG18_FlippedBioFab_RBS (GL1537) using primers oGL1519 and oGL1526; oGL1527 containing P*_tac_* and *lacO*. The resulting plasmid is a pSEVA251-derived vector with the *sfgfp* reporter gene under control of the P*_tac_* promoter. |
| pSEVA251-P*_trc_-sfgfp* | Assembly of the following fragments: vector amplified by PCR from pSEVA251-P*_lac_-sfgfp* using primers oGL1519 and oGL1526; oGL1528 containing P*_trc_* and *lacO*. The resulting plasmid is a pSEVA251-derived vector with the *sfgfp* reporter gene under control of the P*_trc_* promoter. |
| pSEVA251-P*_nptII_-sfgfp* | Assembly of the following fragments: vector amplified by PCR from pBG18 (GL728) using primers oGL291 and oGL1435; P*_nptII_* amplified from pTNV215 (Kaljević *et al.* 2021)  using oGL1436 and oGL452 |
| pSEVA251-P*_lonBb_-sfgfp* | Assembly of the following fragments: vector amplified by PCR from pBG18 (GL728) using primers oGL1351 and oGL1515; P*_lonBb_* amplified from HD100 gDNA (from GL734) using oGL1639 and oGL1640 |
| pSEVA251-P*_dnaKBb_-sfgfp* | Assembly of the following fragments: vector amplified by PCR from pBG18 (GL728) using primers oGL1351 and oGL1515; P*_dnaKBb_* amplified from HD100 gDNA (from GL734) using oGL1641 and oGL1642 |
| pSEVA251-P*_rhoBb_-sfgfp* | Assembly of the following fragments: vector amplified by PCR from pBG18 (GL728) using primers oGL1351 and oGL1515; P*_rhoBb_* amplified from HD100 gDNA (from GL734) using oGL1643 and oGL1644 |
| pSEVA251-P*_lacIq_-sfgfp* | Assembly of the following fragments: vector amplified by PCR from pBG18 (GL728) using primers oGL1078 and oGL1538; P*_lacIq_* promoter amplified from using primers oGL1537 and oGL1352. |
| pSEVA251-P*_nptII_-lacI-*P*_tac_-sfgfp* | Assembly of the following fragments: vector amplified by PCR from pSEVA251-P*_tac_-sfgfp* using primers oGL1792 and oGL1515; *lacI* amplified from pSEVA224 (Martínez-García *et al.* 2014) using primers oGL1790 and oGL1534; P*_nptII_* amplified from pTNV215 (Kaljević *et al.* 2021)  using primers oGL1789 and oGL1791. |
| pK18mobsacB- *bd0063*-P_Biofab_-*sfgfp-bd0064* | Assembly of the following fragments: vector pK18mobsacB amplified by PCR from pK18mobsacB (lab collection) using primers oGL264 and oGL265, UP fragment amplified from HD100 gDNA (GL734) using primers oGL961 and oGL962, P_BioFab_-*sfgfp* amplified from pBG18 (GL728; S. Bigot and C. Lesterlin) using primers oGL963 and oGL964, and fragment DOWN amplified by PCR from HD100 gDNA (GL734) using primers oGL965 and oGL966. |
| pK18mobsacB-*bd0063*-P*_nptII_-lacI-*P*_tac_*-*tdtomato-bd0064* | Assembly of the following fragments: pK18mobsacB_*bd0063*_Up amplified by PCR from pK18mobsacB-*bd0063-*P_Biofab_*-tdtomato-bd0064* (Santin *et al.* 2023) using primers oGL852 and oGL1464; *lacI-*P*_nptII_-P_tac_* (with *lacO*) amplified from pSEVA251-P*_nptII_-lacI-*P*_tac_-sfgfp* using primers oGL2276 and oGL2277; pK18mobsacB_*tdtomato_bd0064*_Down amplified from pK18mobsacB-*bd0063-*P_Biofab_*-tdtomato-bd0064* (Santin *et al.* 2023) using primers oGL1463 and oGL2232. In the resulting construct, *lacI* is under control of constitutive P*_nptII_,* and *tdtomato* is under the control of IPTG-inducible P*_tac_*, with both units in a head-to-head orientation. |
| pK18mobsacB-*bd1853*-P*_dnaKBb_*-*lacI*-P*_tac_-dcas9_Spa_*-*bd1855* | A first construct included the assembly of a vector fragment amplified by PCR from pK18mobsacB-*bd0063-P*_Biofab_*-tdtomato-bd0064* (Santin *et al.* 2023) using primers oGL264 and oGL265; *bd1853_Up* amplified from HD100 gDNA (GL734) using primers oGL1833 and oGL1834; P*_nptII_-lacI-*P*_tac_* (with *lacO*) amplified from pSEVA251-P*_nptII_-lacI-P_tac_-sfgfp* using primers oGL1835 and oGL1836; *bd1855_Down* amplified from HD100 gDNA (GL734) with oGL1839 and oGL1840. This construct served as template for PCR amplification of the vector using oGL1790 and oGL1463, which was used in a subsequent assembly including a P*_dnaKBb_* fragment amplified from pSEVA251-P*_dnaKBb_-sfgfp* using primers oGL2572 and oGL2573. The resulting construct was used for PCR amplification of the pK18mobsacB-*bd1853*-P*_dnaKBb_*-*lacI-*P*_tac_*-*bd1855* vector fragment, which was assembled with a *dcas9_Spa_* fragment amplified from pXGFPC-5-P*_xyl_*-*dcas9_Spa_* ((Guzzo *et al.* 2020); Addgene #133318), hence placing *dcas9_Spa_* under control of the IPTG-inducible P*_tac_*, and *lacI* under control of the constitutive P*_dnaKBb_*, with both units in a head-to-head orientation and flanked by homology regions for their integration at the *bd1853-bd1855* intergenic locus on the *B. bacteriovorus* HD100 chromosome. |
| pK18mobsacB-*bd0063*-P*_dnaKBb_*-*lacI*-*bd0064* | Assembly of the following fragments: vector amplified by PCR from pK18mobsacB-*bd0063-*P_Biofab_*-tdtomato-bd0064* (Santin *et al.* 2023) using primers oGL967 and oGL2232; P*_dnaKBb_*-*lacI* amplified from pK18mobsacB-*bd1853*-P*_dnaKBb_*-*lacI*-P*_tac_-dcas9_Spa_*-*bd1855* using primers oGL2665 and oGL2276. |
| pCRISP-*ftsZ*sgRNA | A first construct assembled the following fragments: pSEVA251-P_Biofab_ vector amplified by PCR from pBG18 (GL728) using primers oGL1964 and oGL1965; anti-*sfgfp* spacer (oGL1966); gRNA_scaffold amplified by PCR from pBXMCS-2-Pconstitutive-sgRNA(Spa)-ctrA ((Guzzo *et al.* 2020); Addgene #133342) using primers oGL1962 and oGL1963. The pCRISP vector was amplified from the resulting construct using primers oGL1965 and oGL1962, and assembled with the *ftsZ_spacer* fragment (oGL2810). |
| pCRISP (pSEVA251-P_Biofab_-Terminator) | Assembly of the following fragments: pCRISP vector amplified by PCR from pCRISP-*ftsZ*sgRNA using primers oGL928 and oGL3126; Fragment amplified from pCRISP-*ftsZ*sgRNA using primers oGL927 and oGL1964. The resulting pCRISP vector does not contain any sgRNA part. |
| pCRISP-I*-bd1075*sgRNA | Assembly of the following fragments: pCRISP-I vector amplified by PCR from pCRISP-I using primers oGL1962 and oGL1965; *bd1075_spacer* fragment (oGL2758). |
| pCRISP-I (pSEVA251-P*_tac_-lacI-*P_Biofab_-Terminator) | A first construct assembled the following fragments: pSEVA251-P_Biofab_ vector amplified by PCR from pBG18 (GL728) using primers oGL1964 and oGL1965; anti-*sfgfp* spacer (oGL1966); gRNA_scaffold amplified by PCR from pBXMCS-2-Pconstitutive-sgRNA(Spa)-ctrA ((Guzzo *et al.* 2020); Addgene #133342) using primers oGL1962 and oGL1963. The resulting construct served as template to amplify a vector fragment by PCR using primers oGL1505 and oGL2098, which was then assembled with the following fragments: *lacI* amplified by PCR from pSEVA224 (Martínez-García *et al.* 2014)  using primers oGL2094 and oGL2095; P*_tac_* fragment (oGL2096). The resulting pCRISP-I vector does not contain any sgRNA part. |
| pSEVA281∆*oriT* | pSEVA281 (Martínez-García *et al.* 2014) in which *oriT* was removed by assembling two fragments amplified by PCR using pSEVA281 as template, with primers oGL303 and oGL695, and oGL286 and oGL696. |

Abbreviations: Kan^R^: kanamycin-resistant; Suc^R^: sucrose-resistant; Kan^S^: kanamycin-sensitive

**Table S4 – Oligos used in this study**

| **Primer name** | **Primer sequence (5’>3’)** |
| --- | --- |
| oGL264 | TCTAGAGTCGACCTGCAG |
| oGL265 | GGATCCCCGGGTACCGAG |
| oGL286 | GGTacccggggatcctcta |
| oGL291 | tcaagagacaggatgaggagaattcATGTCTAAAGGTGAAGAACTGTT |
| oGL303 | gcttgcatgcctgcaggtCGA |
| oGL452 | gaattctcctcatcctgtctct |
| oGL695 | GGCCGGCCccgtagaaaagatca |
| oGL696 | tcttttctacggGGCCGGCCgacaacgcgcggaccgttGTCca |
| oGL852 | ATGGTGAGCAAGGGCGAGGA |
| oGL927 | ATGGCTACCCATAAGCCTATCA |
| oGL928 | TGATAGGCTTATGGGTAGCCAT |
| oGL961 | TACGAATTCGAGCTCGGTACCCGGGGATCCatcagaactgtttccaaaaaccttctgg |
| oGL962 | atactctatcgatgcctcgctcttttcttttttggagaaccc |
| oGL963 | aaagaaaagagcgaggcatcgatagagtattgacttcgc |
| oGL964 | aagagcgagtgaaaaTTATTTGTAGAGCTCATCCATGCCG |
| oGL965 | GAGCTCTACAAATAAttttcactcgctctttttgtttttgcg |
| oGL966 | AAGCTTGCATGCCTGCAGGTCGACTCTAGAagattgttcataatgttcgagggtttaacac |
| oGL967 | ttttcactcgctctttttgtttttgcg |
| oGL1078 | TCTAGAGGATCCCCGGGT |
| oGL1351 | ATGTCTAAAGGTGAAGAACTGTTCACCG |
| oGL1352 | ATTCACCACCCTGAATTGACTCTCT |
| oGL1435 | TCTAGAGGATCCCCGGGT |
| oGL1436 | GCTCGGTACCCGGGGATCCTCTAGAacgctgccgcaagcact |
| oGL1463 | GGATCTCGTCGTGACCCATGG |
| oGL1464 | ccatgggtcacgacgagatcc |
| oGL1505 | TCTAGAGGATCCCCGGGTACC |
| oGL1515 | atgcgtcgactctagagga |
| oGL1516 | CAGTTCTTCACCTTTAGACATtaaaaaacctccttaGAGCTCcgatagagtattgacttcgca |
| oGL1517 | ctctagagtcgacgcatATTCACCACCCTGAATTGACT |
| oGL1518 | taaggaggttttttaATGTCTAAAGGTGAAG |
| oGL1519 | GAGCTCcgatagagtattgacttcg |
| oGL1524 | caatactctatcgGAGCTCTTTACACTTTATGCTTCCGGC |
| oGL1525 | CTTTAGACATtaaaaaacctccttaTGTGAAATTGTTATCCGCTCACA |
| oGL1526 | TGTGGAATTGTGAGCGGA |
| oGL1527 | caatactctatcgGAGCTCttgacaattaatcatcggctcgtataatgTGTGGAATTGTGAGCG |
| oGL1528 | caatactctatcgGAGCTCttgacaattaatcatccggctcgtataatgTGTGGAATTGTGAGCG |
| oGL1534 | ctctagagtcgacgcattcactgcccgctttcca |
| oGL1537 | gacaccatcgaatggtgcaa |
| oGL1538 | tttgcaccattcgatggtgtctctagaggatccccGGGT |
| oGL1641 | tcctctagagtcgacgcatctgcctcataacacgagttaaaacc |
| oGL1642 | CGGTGAACAGTTCTTCACCTTTAGACATacctaaatctccttaaaattagctgttttc |
| oGL1643 | CGGTGAACAGTTCTTCACCTTTAGACATacaaattctccttgtaggattt |
| oGL1644 | tcctctagagtcgacgcatgacaggcaaggggacctg |
| oGL1789 | acgctgccgcaagca |
| oGL1790 | gtgaaaccagtaacgttatacgatg |
| oGL1791 | taacgttactggtttcacgaattctcctcatcctgtctct |
| oGL1792 | tgcttgcggcagcgtGAGCTCttgacaattaatcatcgg |
| oGL1833 | CTCGGTACCCGGGGATCCccgacggaagaatctgttcg |
| oGL1834 | ctggaaagcgggcagtgaaacgcattattgccagaaaaaac |
| oGL1835 | tcactgcccgctttccag |
| oGL1836 | cgatgctgtacttcttgtccattaaaaaacctccttaTGTGAAAT |
| oGL1839 | ctcagctgggaggcgacTAAagaagctcaaagggctgc |
| oGL1840 | CTGCAGGTCGACTCTAGAgagctgtttgatgtcctgattcg |
| oGL1962 | gtttttgtactcgaaagagcct |
| oGL1963 | TCAACAGGAGTCcaaaaaaaagcacctcatgtcaaaaca |
| oGL1964 | ttttgGACTCCTGTTGATAGATCC |
| oGL1965 | atgaatctattataggtacaaaaagatgcg |
| oGL1966 | tttttgtacctataatagattcatAACCAGGATCGGAACAACACgtttttgtactcgaaagag |
| oGL2094 | GGTACCCggggatcctctagatcactgcccgctttccag |
| oGL2095 | TGTTCACAtaaggaggttttttagtgaaaccagtaacgttatacgatg |
| oGL2096 | taaaaaacctccttaTGTGAACAcattatacgagccgatgattaattgtcaa |
| oGL2098 | gagccgatgattaattgtcaagtcgacgcatcgatagagta |
| oGL2232 | CTCGCTCTTTTCTTTTTTGGAGAACCC |
| oGL2276 | caaaaaagaaaagagcgagtcactgcccgctttccag |
| oGL2277 | GCCCTTGCTCACCATtaaaaaacctccttaTGTGAAATTGTTATCC |
| oGL2572 | gatgattaattgtcaaGAGCTCctgcctcataacacgagttaaaacc |
| oGL2573 | gtataacgttactggtttcacacctaaatctccttaaaattagctgttttc |
| oGL2665 | cgcaaaaacaaaaagagcgagtgaaaactgcctcataacacgagttaaaacc |
| oGL2758 | ttgtacctataatagattcatcaacgcaaataaagcacagcgtttttgtactcgaaagag |
| oGL3126 | TCTATCAACAGGAGTCcaaaaatgaatctattataggtacaaaaagatgcg |

**References**

Gil D, Bouche JP. ColE1-type vectors with fully repressible replication. *Gene* 1991;**105**:17–22.

Guzzo M, Castro LK, Reisch CR *et al.* A CRISPR Interference System for Efficient and Rapid Gene Knockdown in Caulobacter crescentus. Goley ED, Komeili A (eds.). *mBio* 2020;**11**:744.

Kaljević J, Saaki TNV, Govers SK *et al.* Chromosome choreography during the non-binary cell cycle of a predatory bacterium. *Curr Biol* 2021;**31**:3707-3720.e5.

Martínez-García E, Aparicio T, Goñi-Moreno A *et al.* SEVA 2.0: an update of the Standard European Vector Architecture for de-/re-construction of bacterial functionalities. *Nucleic Acids Res* 2014;**43**:D1183–9.

Rendulic S, Jagtap P, Rosinus A *et al.* A Predator Unmasked: Life Cycle of Bdellovibrio bacteriovorus from a Genomic Perspective. *Science* 2004;**303**:689–92.

Santin YG, Lamot T, Raaphorst R van *et al.* Modulation of prey size reveals adaptability and robustness in the cell cycle of an intracellular predator. *Curr Biol* 2023;**33**:2213-2222.e4.
