# Supplementary figures and images for: Expanding the genetic toolbox of the obligate predatory bacterium Bdellovibrio bacteriovorus with inducible gene expression and CRISPR interference
Source: Microlife. 2025 Sep 1;6:uqaf021. doi: 10.1093/femsml/uqaf021 (PMC12448681; doi:10.1093/femsml/uqaf021)

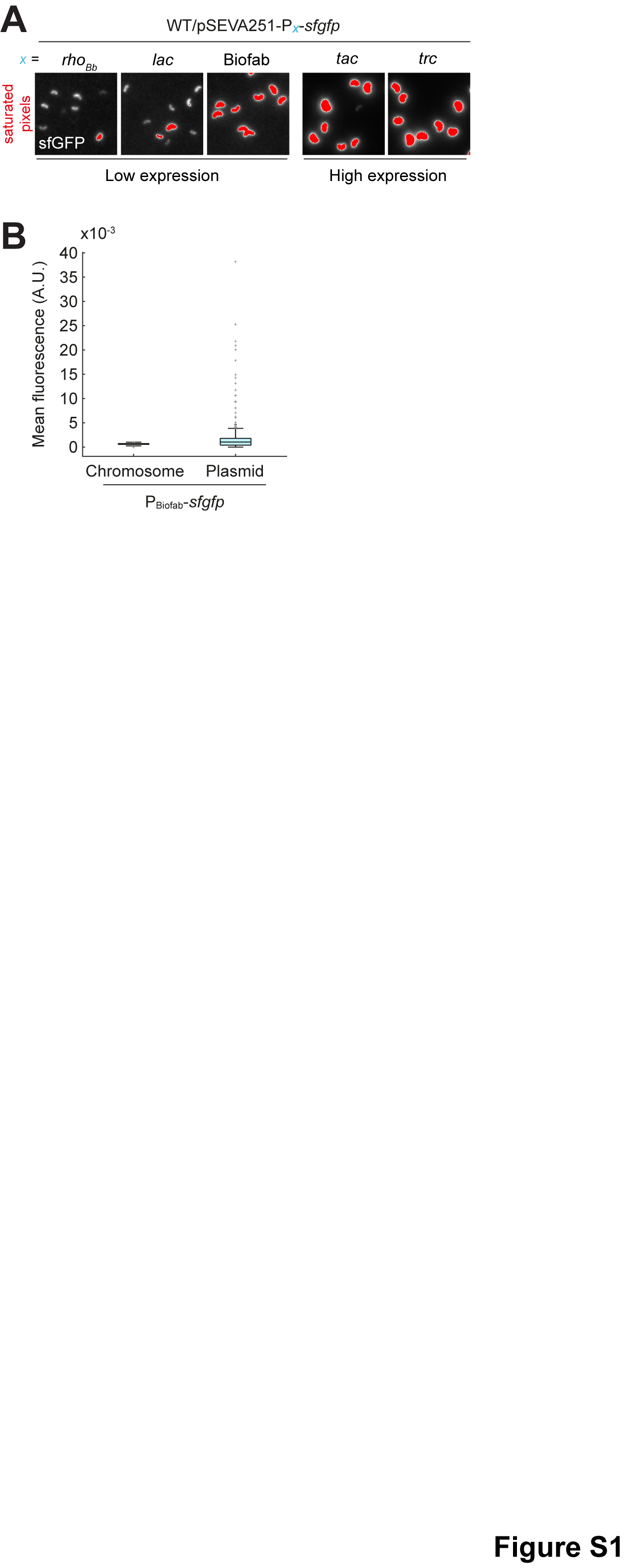

Supplement: uqaf021_Supplemental_Files [file uqaf021_supplemental_files.zip › R1_Figure S1_de Pierpont et al.tif]

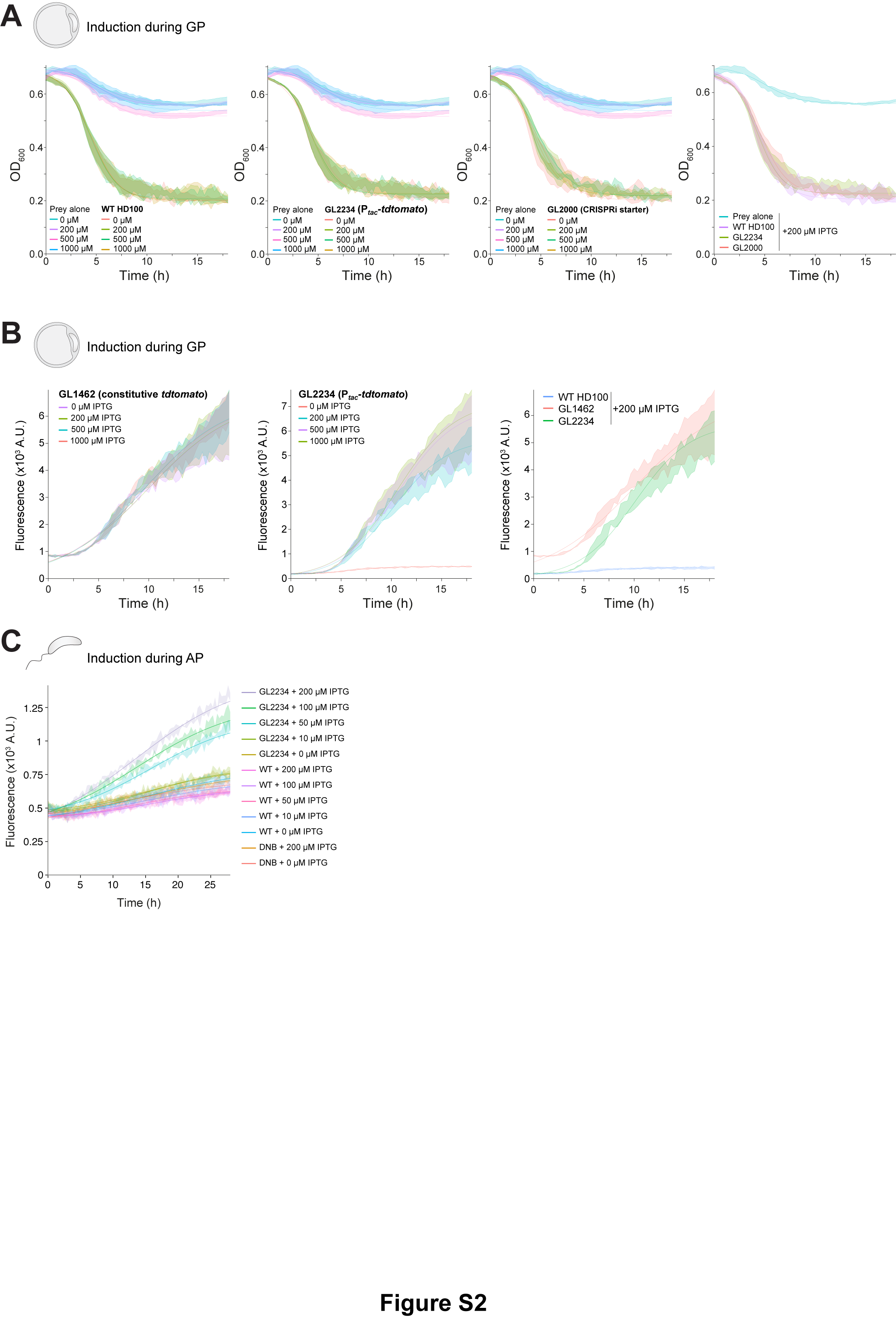

Supplement: uqaf021_Supplemental_Files [file uqaf021_supplemental_files.zip › R1_Figure S2_de Pierpont et al.tif]
